# Hip Replacement as Alternative to Intramedullary Nail in Elderly Patients with Unstable Intertrochanteric Fracture: A Systematic Review and Meta‐Analysis

**DOI:** 10.1111/os.12532

**Published:** 2019-10-29

**Authors:** Jia‐bao Ju, Pei‐xun Zhang, Bao‐guo Jiang

**Affiliations:** ^1^ Department of Orthopedics and Trauma Peking University People's Hospital Beijing China

**Keywords:** hip arthroplasty, intertrochanteric fractures, intramedullary nail

## Abstract

**Objective:**

To evaluate the efficacy and safety of hip replacement and intramedullary nails for treating unstable intertrochanteric fractures in elderly patients.

**Methods:**

Randomized clinical trials (RCTs) to compare hip replacement with intramedullary nail in the management of elderly patients with unstable intertrochanteric femur fracture were retrieved from Cochrane Library (up to January 2018), CNKI (China National Knowledge Infrastructure), Wanfang Data, PubMed, and Embase. The methodological quality of the included trials was assessed using the Cochrane risk of bias assessment tool, and relevant data was extracted. Statistical analysis was performed by Revman 5.3. Where possible, we performed the limited pooling of data.

**Results:**

Fourteen trials including a total of 1067 participants aged 65 and above were included for qualitative synthesis and meta‐analysis. The methodological quality of the included study was poor. The meta‐analysis indicated that the hip replacement group benefited more than the intramedullary nail group in terms of the bearing load time (WMD ‐14.61, 95% CI −21.51 to −7.7, *P* < 0.0001), mechanical complications (OR 0.34, 95% CI 0.21 to 0.57, *P* < 0.0001), and post‐operative complications (OR 0.46, 95% CI 0.22 to 0.93, *P* = 0.03). While the intramedullary nail was superior to arthroplasty regarding the intraoperative blood loss (WMD 58.36, 95% CI 30.77 to 85.94, *P* < 0.0001). However, there were no statistical significances in the length of surgery (WMD 5.27, 95% CI 4.23 to 14.77, *P* = 0.28), units of blood transfusion (WMD 0.34, 95% CI ‐0.16 to 0.85, *P* = 0.18), length of hospital stay (WMD ‐1.00, 95% CI ‐2.93 to 0.93, *P* = 0.31), Harris hip score (WMD 0.31, 95% CI ‐0.39 to 1.01, *P* = 0.38), and mortality (OR 1.24, 95% CI 0.12 to 13.10, *P* = 0.86).

**Conclusions:**

This systematic review and meta‐analysis provided evidence for the efficacy and safety of hip replacement and intramedullary nail in treating unstable intertrochanteric fractures. However, the results should be interpreted cautiously because of methodological limitations and publication bias.

## Introduction

Hip fracture is the most common fracture in elderly patients, with the rate of morbidity increasing with this issue.^1^ The functional outcomes are influenced by many factors, including patient condition, type of fracture, and therapeutic implants. It is estimated that few patients could return to the premorbid functional status. Besides, limitation of motion brings huge economic burdens to health care insurance.^2^

Referring to the hip capsule, hip fracture can be classified as intra‐capsular fracture (femoral neck fracture) and extra‐capsular fracture (intertrochanteric fracture), of which intertrochanteric fractures can be further classified as 31A1, 31A2, and 31A3 types according to AO/OTA classification,^3^ and type A2 and A3 represented unstable fractures. Besides, the intertrochanteric fracture could also be subdivided into type I, II, III, IV, and V according to Evans‐Jensen classification,^4^ and type III, IV, and V are considered as unstable type.

Based on evidence from clinical trials, surgical treatments allow early rehabilitation and functional recovery, which could remarkably decrease the associated mortality and medical complications.^5^ Furthermore, Simunovic *et al*. found early surgery within 72 h of the event was associated with a lower risk of death and lower rates of post‐operative complication.^6^

The American Academy of Orthopedic Surgeons (AAOS) recommends dynamic hip screw and intramedullary nail as the main surgery options in stable intertrochanteric fracture.^7^ For unstable type, compared with the dynamic hip screw group, intramedullary nail could improve mobility and quality of life of patients.^8^ However, intramedullary nail may fail in unstable intertrochanteric fractures, and hip replacement could be used as an alternative.^9^

Several systematic reviews and meta‐analyses have tested effects of hip replacement and intramedullary nail in the treatment of intertrochanteric fracture. Parker *et al*. pooled data from two randomized clinical trials including a total of 148 people aged 70 years or over with unstable intertrochanteric fractures. Due to disparities in the interventions between groups and small sample sizes, there was insufficient evidence to detect any clear differences in outcomes except for higher numbers of patients in the arthroplasty group requiring blood transfusion.^10^ Another newly published systematic review had revealed that compared to intramedullary nail fixation, the use of arthroplasty could reduce the implant‐related complications and reoperations rates. But there were significant differences in terms of duration of surgery, blood loss, transfusion requirement, hip joint function, and mortality at 1 year in favor of intramedullary nail group.^11^ However, the intertrochanteric fractures in this review were not specific to unstable type. The optimal treatment for unstable intertrochanteric fractures is still controversial. Thus, the purpose of this meta‐analysis is to evaluate the efficacy and safety of hip replacement and intramedullary nail in the treatment of unstable intertrochanteric fracture in elderly patients and to provide the best available evidence of clinical practice.

## Methods

### *Electronic Searches*

We electronically searched the Cochrane Library (Issue February 2018), PubMed (January 2000 to December 2017), Embase (January 2000 to December 2017), CNKI Database (January 2000 to December 2017), and Wanfang Database (January 2000 to December 2017). We used a specific set of mesh terms and free terms to search the database: (intertrochanteric femur fracture OR femoral intertrochanteric fracture OR trochanteric fracture OR extra‐capsular hip fracture) AND (hip replacement OR hip arthroplasty OR hemi‐arthroplasty OR total hip replacement OR internal fixation OR cephalomedullary nail OR intramedullary nail). We also searched the reference lists of included studies to identify additional eligible trials.

### *Inclusion Criteria*

#### *Types of Studies*

We included all RCTs comparing hip replacement with intramedullary nail. All eligible trials published in Chinese or English were included.

#### *Types of Participants*

We considered trials that included elderly patients aged 65 and above with unstable intertrochanteric fractures. Unstable fractures in the femoral trochanter region should be confirmed with anteroposterior (AP) and lateral radiographs, CT or MRI based on the AO or Evens‐Jensen classifications.

#### *Types of Interventions*

Participants were treated with surgery of hip replacement (hemi‐arthroplasty or total hip arthroplasty (THA)) and intramedullary nail, such as the proximal femoral nail anti‐rotation, proximal femoral nail, or Gamma‐3 nail. And trials reported at least one outcome measure.

#### *Types of Outcomes*

We included studies assessing at least one quantitative measurement (length of surgery, blood loss, units of transfusion, bearing load time, hospital stay, Harris hip score), mortality, and complications (mechanical complications and post‐operative complications).

### *Exclusion Criteria*

Exclusion criteria included: (i) lack of quantitative calculation on outcomes of interest; (ii) data was duplicated or overlapped; and (iii) insufficient assessment of methodological quality.

### *Outcome Assessments*

Data on the following outcomes were collected: (i) operative details: length of surgery (in minutes), operative blood loss (in milliliters), perioperative blood transfusion (in units); (ii) mechanical complications: operative fracture of the femur, periprosthetic fracture, reoperation; (iii) complications specific to hip replacement: dislocation, acetabular wear, loosening of the prosthesis; (iv) complications specific to intramedullary nail: cutout of the implant proximally, non‐union of the fracture, avascular necrosis of the femoral head; (v) post‐operative complications: superficial wound infection, deep wound infection, pneumonia, venous thromboembolism (deep vein thrombosis or pulmonary embolism), urinary tract infection, neurological complication, any medical complications; (vi) hospital stay: length of hospital stay (in days); (vii) bearing load time: time to bear partial or full weight (in days); (viii) final outcome measures: functional outcome assessment (Harris hip score at final follow up); and (ix) mortality (within the follow‐up period of the study).

### *Data Collection and Analysis*

Both authors independently scanned the titles and abstracts of articles derived from the search and kept all potentially relevant articles. Both authors performed data extraction of included trials and any discrepancies were resolved through discussion. We extracted the following data from the included trials: authors, publication date, study design, participant characteristics, intervention, follow‐up period, and outcomes. The outcomes pooled in the analysis included length of surgery, perioperative blood loss, units of blood transfusion, mechanical complications, postoperative complications, bearing load time, hospital stay, Harris hip score, and mortality.

### *Risk of Bias Assessment in Included Trials*

Two review authors independently used the risk of bias assessment tool in the Cochrane handbook for the systematic reviews of interventions to assess the methodological quality of each included trial.^12^ The specific domains were assessed as follows: random sequence generation, allocation sequence concealment, blinding, incomplete outcome, selective outcome reporting, and other sources of bias. We graded the risk of bias for each domain as low risk of bias, high risk of bias, or unclear. We settled assessment disagreements by discussion.

### *Data Synthesis*

For individual trials, we report the odds ratio (OR) with 95% confidence intervals for dichotomous outcomes, and weighted mean differences (MD) with 95% confidence intervals for continuous outcomes. Results of included trials were pooled using both the fixed‐effects and random‐effects models. Heterogeneity between comparable trials was tested using a standard chi^2^ test, with additional consideration of the I^2^ statistic. If we decided to pool the results under the statistically significant heterogeneity (*P* < 0.1, I^2^ > 50%), we presented the results for the random‐effects model.

### *Patient and Public Involvement*

Neither patients nor public were involved in this study.

## Results

### *Study Selection*

**Figure 1:**
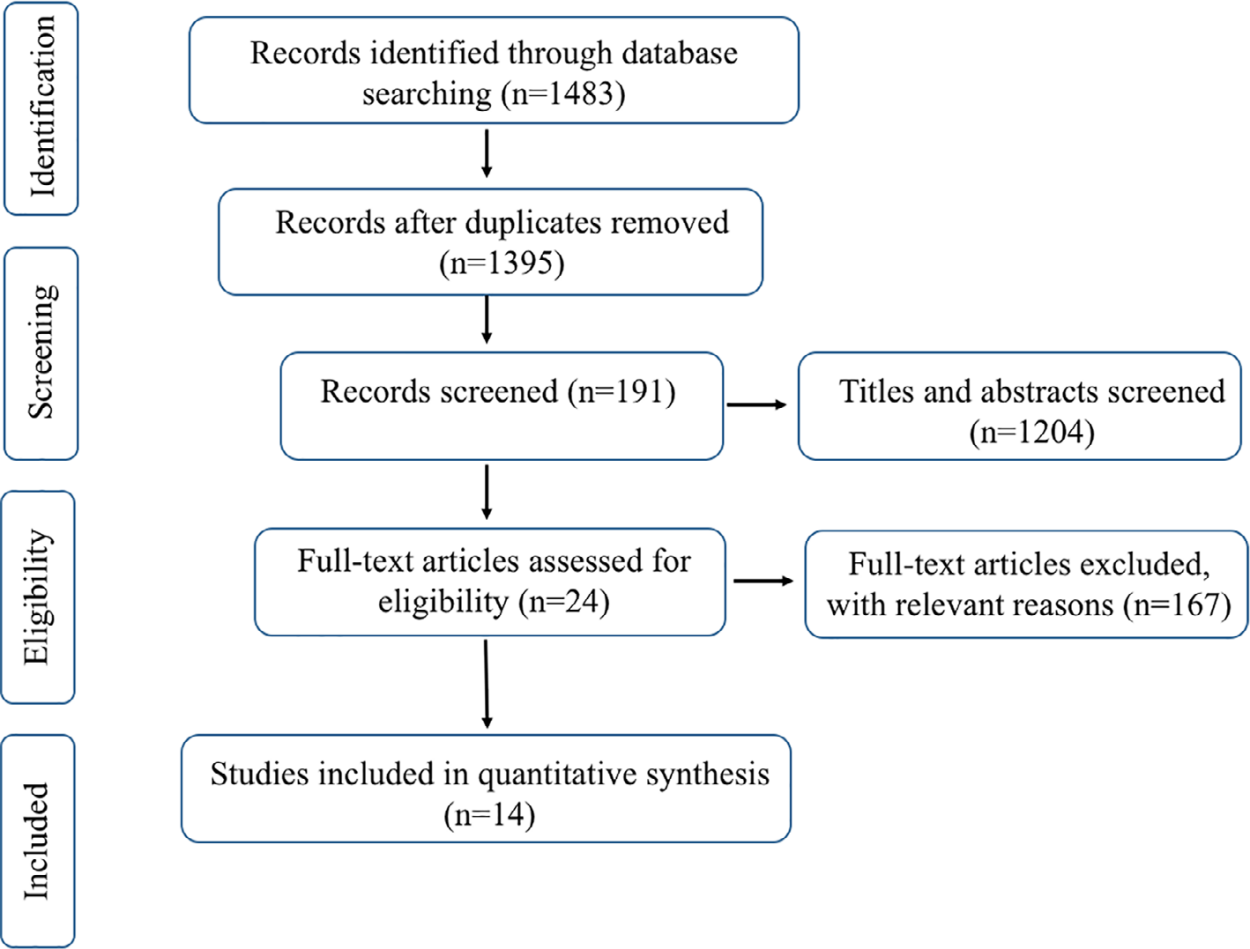
Study flow diagram. CNKI, Chinese National Knowledge Infrastructure. HR, Hip Replacement. RCTs, Randomized Clinical Trials.

We initially identified 1483 titles and abstracts through the primary search in databases. After excluding duplications and mismatches, 24 full‐text articles were downloaded to assess for eligibility. In the end, 14 trials met the eligibility criteria and were included in our research. The search flow was described in detail in Fig. 1.

### *Study Characteristics*

Studies enrolled in the review were published between 2005 and 2017. A total of 1067 elderly patients from 14 trials were included. Two trials were published in English, and the other trials were published in Chinese. The sample size ranged from 38 to 126, and the average age of patients ranged from 65 to 86 years. Fracture type in 11 trials was based on Evans‐Jensen classification. In intramedullary nail fixation group, 194 patients underwent proximal femoral nail and 340 patients received proximal femoral nail anti‐rotation, while 533 patients underwent hemi‐arthroplasty in hip replacement group. The follow‐up time ranged from 3 to 48 months. A comprehensive characteristic of the trials included in this meta‐analysis were reported in Table 1 9, 13, 14, 15, 16, 17, 18, 19, 20, 21, 22, 23, 24, 25.

**Table 1:** Characteristics of included trials

Study ID	Sample size		Fracture classification		Age (year)		Sex (male/female)		Intervention		Follow‐up (month)	
	HR	IN	HR	IN	HR	IN	HR	IN	HR	IN	HR	IN
Chen 2012^13^	19	19	III:18		76.1		26/12		HA	PFNA	6	6
			IV:20									
Deng 2016^14^	30	30	III:17	III:18	85 ± 4.4	84 ± 3.6	NR	NR	HA	PFNA	12	12
			IV:9	IV:8								
			V:4	V:4								
Desteli 2015^15^	42	44	A1.3:6	A1.3:6	65 ± 1.52	67 ± 1.72	26/16	27/15	HA	PFNA	24	24
			A2.2:18	A2.2:18								
			A2.3:10	A2.3:10								
			A3.1:6	A3.1:6								
			A3.3:4	A3.3:4								
Guo 2017^16^	40	40	III:80		78.5 ± 7.1	77.3 ± 6.2	25/15	22/18	HA	PFNA	6	6
Kim 2005^9^	29	29	A2:29	A2:20	82 ± 3.4	81 ± 3.2	6/23	8/21	HA	PFN	48	48
Li 2015^17^	25	25	III:14	III:15	76.64 ± 1.32	75.01 ± 1.93	10/15	11/14	HA	PFNA	12	12
			IV:11	IV:10								
Su 2016^18^	45	45	IIIa:20	III:21	73 ± 2.62	75 ± 3.12	25/20	28/17	HA	PFNA	12	12
			IIIb:10	IIIb:9								
			IV:15	IV:15								
Wang 2012^19^	30	33	III:39		78.2		28/35		HA	PFNA	12	12
			IV:20									
			V:4									
Wang 2016^20^	24	24	III:15	III:17	85.6 ± 2.4	85.9 ± 1.8	5/19	7/17	HA	PFN	NR	NR
			IV:4	IV:3								
			V:5	V:4								
Wang 2017^21^	39	39	IIIa:16	IIIa:16	79.6 ± 1.3	76.8 ± 1.4	21/18	20/19	HA	PFN	NR	NR
			IIIb:14	IIIb:13								
			IV:7	IV:8								
			V:2	V:2								
Xu 2017^22^	39	39	A2:29	A2:28	78.3 ± 4.6	78.5 ± 5.7	18/21	17/22	HA	PFN	NR	NR
			A3:10	A3:11								
Ye 2016^23^	46	46	III:30		70.6 ± 3.2		50/42		HA	PFNA	3	3
			IV:36									
			V:26									
Zhao 2016^24^	60	60	IIIa:33	IIIa:35	80.2 ± 4.3	79.2 ± 5.3	30/30	32/28	HA	PFNA	3	3
			IIIb:20	IIIb:19								
			IV:6	IV:5								
			V:1	V:1								
Zhu 2016^25^	63	63	IIIa:20	IIIa:19	82.8 ± 3.1	81.5 ± 2.6	25/38	23/40	HA	PFN	NR	NR
			IIIb:20	IIIb:21								
			IV:23	IV:23								

HR, hip replacement; IN, intramedullary nail; HA, hemi‐arthroplasty; PFN, proximal femoral nail; PFNA, proximal femoral nail anti‐rotation; NR, not referred.

### *Assessing Risk of Bias in Included Studies*

**Figure 2:**
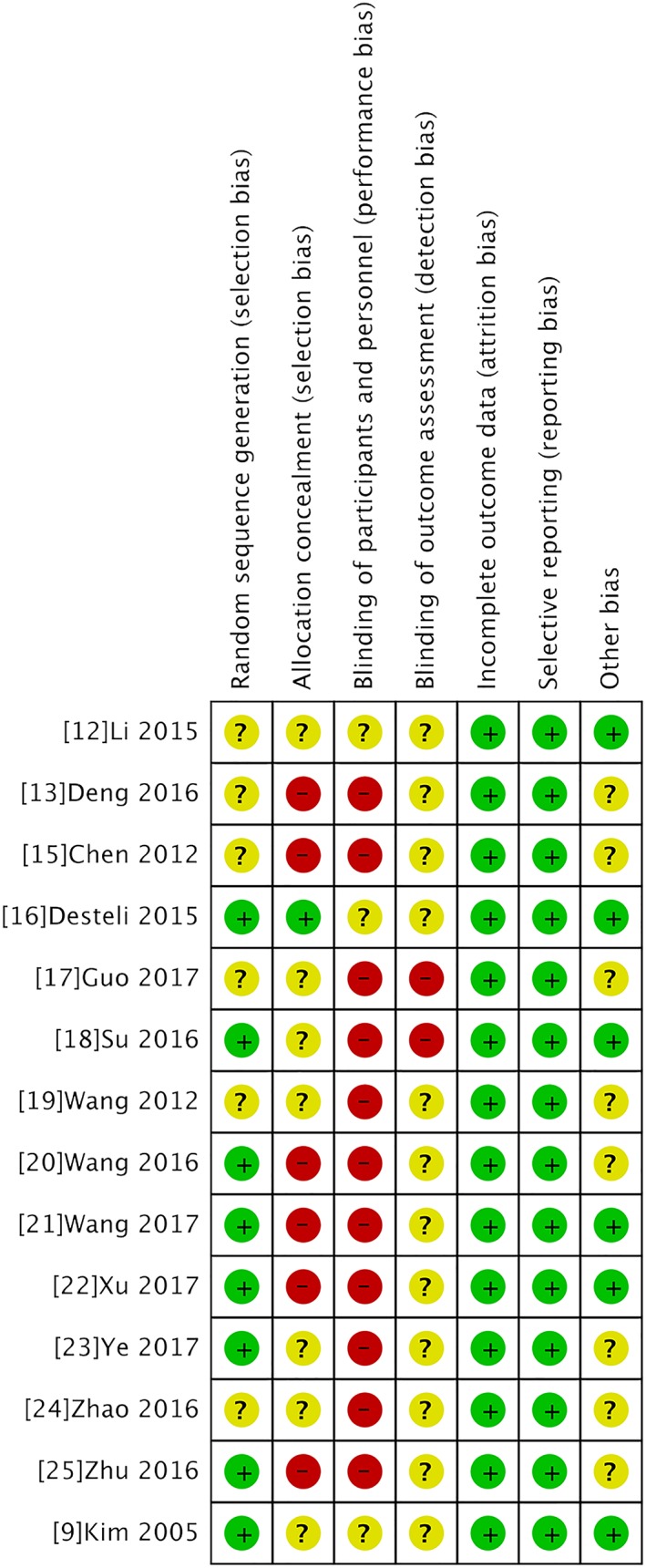
Risk of bias assessment of included trials.

The results of the methodological assessment for individual trials were given below. In random sequence generation, eight trials used proper methods with a low risk of bias, and the random number sequences were produced by either random number tables or computer software. These trials had not described the specific steps and methods of statistical analysis. The results of the assessment are presented in Fig. 2. It is virtually impossible to blind the surgeons and patients to surgeries, and none of the included RCTs reported blinding of the surgeons, participants, or assessors.

### *Length of Surgery*

**Figure 3:**
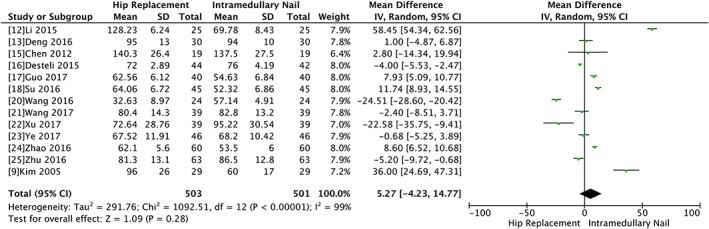
Forest plot and meta‐analysis of length of surgery.

Thirteen trials provided data on duration of surgery, involving 1004 fractures. The pooled results indicated that there was no statistical difference in operation time between the two groups (WMD 5.27, 95% CI ‐4.23 to 14.77, *P* = 0.28) with significant heterogeneity (Chi^2^ = 1092.51, *P* < 0.00001, I^2^ = 99%). However, the result of sensitivity by excluding the outlier study did not alter significance, suggesting the result was reliable (Fig. 3).

### *Blood Loss*

**Figure 4:**
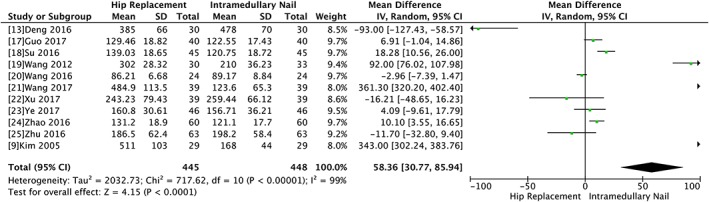
Forest plot and meta‐analysis of blood loss.

There were 12 articles involving 943 fractures that provided data of operative blood loss. The heterogeneity test indicated there was statistical heterogeneity (Chi^2^ = 13194.19, *P* < 0.00001, I^2^ = 100%) and the outcome showed there was no significant difference between the hip replacement and intramedullary nail group (WMD 83.97, 95% CI −5.34 to 173.28, *P* = 0.07). The result of sensitivity showed if Li 2015 was excluded, there was a significant difference between the two groups (WMD 58.36, 95% CI 30.77 to 85.94, *P* < 0.0001) (Fig. 4).

### *Units of Blood Transfusion*

**Figure 5:**
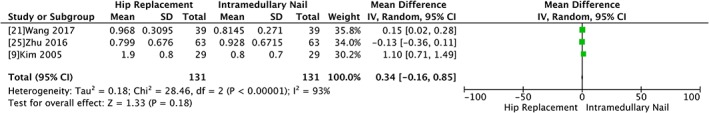
Forest plot and meta‐analysis of units of transfusion.

Units of transfusion were documented in three articles. The pooled data indicated transfusion requirements showed no significant difference between the two intervention groups (WMD 0.34, 95% CI ‐0.16 to 0.85, *P* = 0.18) with significant heterogeneity (Chi^2^ = 28.46, *P* < 0.00001, I^2^ = 93%). The result of sensitivity showed the result would not be changed when the outlier result was excluded (Fig. 5).

### *Mechanical Complications*

**Figure 6:**
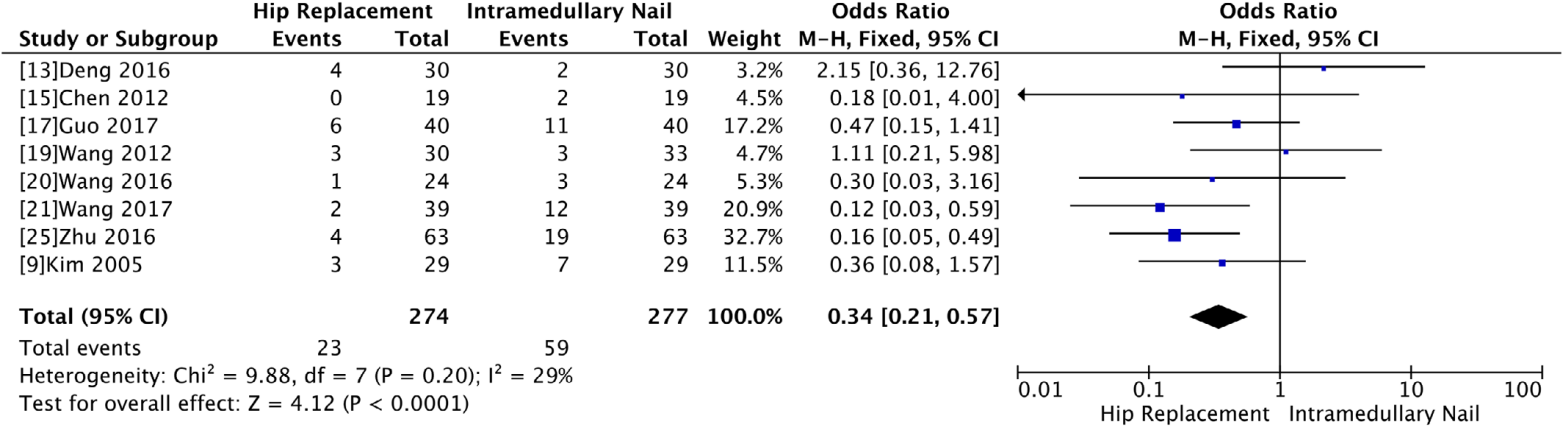
Forest plot and meta‐analysis of mechanical complications.

The mechanical complications were evaluated in nine trials with 551 participants. There was significant difference between hip replacement and intramedullary nail groups (OR 0.34, 95% CI 0.21 to 0.57, *P* < 0.0001) without significant heterogeneity (Chi^2^ = 9.88, *P* = 0.2, I^2^ = 29%) (Fig. 6).

### *Post‐operative Complications*

**Figure 7:**
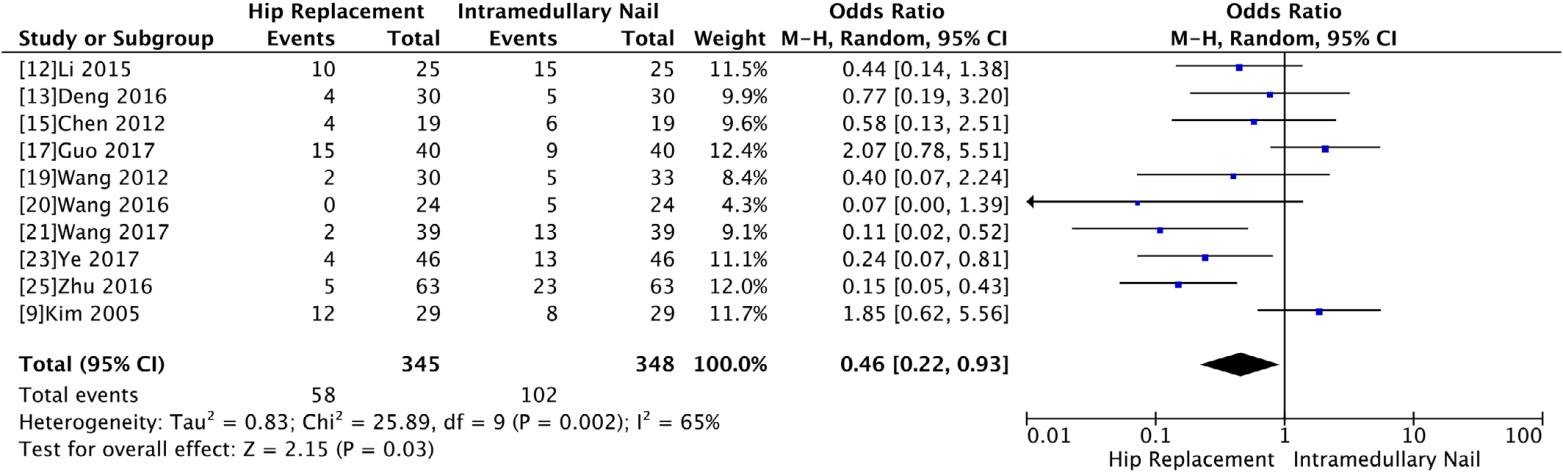
Forest plot and meta‐analysis of post‐operative complications.

Eleven trials with a total of 693 participants showed significant difference regarding post‐operative complications between the two groups (OR 0.46, 95% CI 0.22 to 0.93, *P* = 0.03) with significant heterogeneity (Chi^2^ = 25.98, *P* = 0.002, I^2^ = 65%) (Fig. 7).

### *Bearing Load Time*

**Figure 8:**
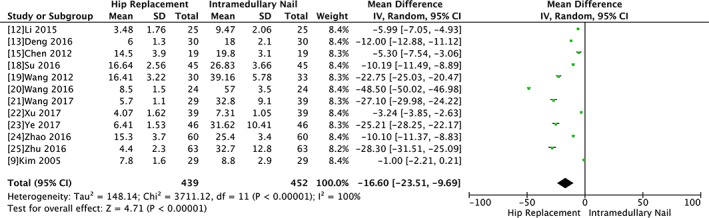
Forest plot and meta‐analysis of bearing load time.

The bearing load time between the two groups was evaluated by a random model due to significant heterogeneity (Chi^2^ = 3711.12, *P* < 0.00001, I^2^ = 100%). However, there was significant difference between the two groups in terms of time to bear weight (WMD ‐16.6, 95% CI ‐23.51 to −9.69, *P* < 0.00001). The sensitivity analysis showed there was no significant change when any study was omitted (Fig. 8).

### *Hospital Stay*

**Figure 9:**
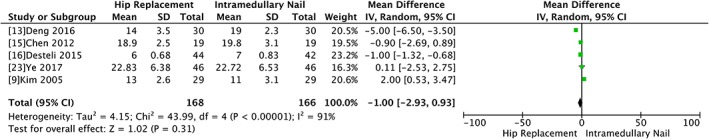
Forest plot and meta‐analysis of length of hospital stay.

The hospital stay was documented in three articles. The pool data indicated there was no significant difference between the two groups (WMD ‐1.0, 95% CI ‐2.93 to 0.93, *P* = 0.31) with significant heterogeneity (Chi^2^ = 43.99, *P* < 0.00001, I^2^ = 91%). We used the method of removing study by study to test the stability, and the results showed there had been no noticeable change (Fig. 9).

### *Harris Hip Score*

**Figure 10:**
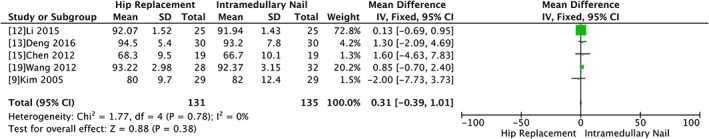
Forest plot and meta‐analysis of the Harris hip score.

Five trials involving 266 participants used the Harris hip score to evaluate the functional outcome. The mean Harris hip score at final follow‐up between the two groups was evaluated by a fixed‐model, which showed there was no significant difference (WMD 0.31, 95% CI ‐0.39 to 1.01, *P* = 0.38) without any heterogeneity (Chi^2^ = 1.77, *P* = 0.78, I^2^ = 0%) (Fig. 10).

### *Mortality*

**Figure 11:**
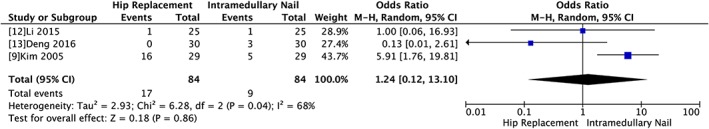
Forest plot and meta‐analysis of mortality.

Three trials with a total of 168 participants showed that there was no significant difference in mortality between hip replacement and intramedullary nail group (OR 1.24, 95% CI 0.12 to 13.10, *P* = 0.86) with significant heterogeneity (Chi^2^ = 6.28, *P* = 0.04, I^2^ = 68%) (Fig. 11).

### *Sensitivity Analysis*

We used the method of removing study by study to test the stability the meta‐analysis, and the results showed there had been no significant change on any of the outcomes except the post‐operative complications. When using different statistical models to pool the data for the post‐operative complications, we found observable change, which indicated the result was not solid.

## Discussion

The goal of care for elderly adults with unstable intertrochanteric fracture is to stabilize the fracture to achieve immediate pain relief, rapid mobilization, and accelerated rehabilitation to self‐maintenance. The intramedullary nail is the commonly used treatment for intertrochanteric fracture. However, it may fail in unstable type. Some surgeons proposed to choose the hip replacement as the primary treatment, especially in elderly patients with low bone mineral density or osteoporosis, to decrease the reoperation and implant‐related complications despite not having sufficient evidence.

Our study showed the hip replacement could shorten the time to immobilize notably compared with intramedullary nail, but there is a significant heterogeneity, which could be explained by the rigidity of different implants and differences between cement and cementless arthroplasty. Konstantinidis reported in his biomechanical study that in osteoporotic bone proximal femoral nail could withstand 400 cycles of load at 2100 N, and in healthy bone it could bear the same load for 20,000 cycles.^26^ Biomechanical studies have shown that InterTAN is almost twice as strong as cephalomedullary nails with load to 8000 N at the central position and 6,000 N for decentralized position.^27^ The reported torque resistance was also high at around 3.8 newton/m. Besides, for most of medical centers in China, surgeons usually advise patients to avoid bearing load at least 4 weeks after intramedullary nail fixation until the callus is formed at the fracture site. Compared with osteosynthesis, patients treated with hip replacement are advised to bear partial weight 3 days later after surgery. Notably, variations of perioperative management and rehabilitation strategies between medical centers contributed to the heterogeneity.

For blood loss, there was a statistical difference between the two groups in favor of intramedullary nail with obvious heterogeneity, which is similar to the result from a previous study.^11^ Various methods to calculate the blood loss and different levels of expertise of the surgeons in those trials were sources of heterogeneity. With rapid improvements of the fixation devices, the surgery time became shorter and intraoperative blood loss was reduced. Notably, Kim *et al*.^9^ reported a larger amount of blood loss than the other trials did, and the difference may be derived from different devices and levels of expertise of surgeons.

In our study, compared with hip replacement, the intramedullary nail has a higher mechanical or implant‐related complication rate. Common problems in intramedullary nail were cutout of the implant, non‐union of the fracture, and avascular necrosis of the femoral head, while dislocation was the major concern of the hip replacement. Proximal femoral nail antirotation (PFNA) has a proximal blade instead of lag screw, which is different from proximal femoral nail. The blade was inserted by impaction, thereby avoiding rotation of femoral head and compaction of bone around the blade to reduce the risk of cutout.^28^ Nevertheless, Simmermacher *et al*. reported six cutouts and periprosthetic fractures in seven patients of 315 patients with unstable intertrochanteric fractures treated with PFNA.^29^ In our study, there are in total 59 events in intramedullary nail group and 23 events in hip replacement, and the most common mechanical complication is cutout. Despite the modified design of the fixation devices, there is continuing concern of cutout. Yu *et al*. included 29 trials reporting cutout data in their meta‐analysis.^30^ The pooling of the trials showed gama nail increased the risk of cutout compared to sliding hip screw, and no significant differences were found in PFN or PFNA comparisons.

The meta‐analysis showed the hip replacement could obviously reduce post‐operative complications. Frequent complications are pressure sores, pneumonia, urinary tract infection, and deep vein thrombosis, and all these complications are due to long‐term immobilization. However, Gormeli *et al*.^31^ reported no statistically significant differences between the hemiarthroplasty and proximal femoral nail in medical complications, which is similar to the data published by Parker.^10^ The different complication rates have contributed to different perioperative managements among medical centers. The patients were usually mobilized to bear partial or restricted weight on the day following intramedullary nail surgery in Europe and the United States. However, immobilization extended to 4 weeks in China. Effective prevention for medical complications is to allow the patients to ambulate early. The patients treated with hip replacement are allowed to move around much earlier than those treated with intramedullary nail, which could reduce the medical complications rate significantly.

The meta‐analysis showed there was no significant difference between the hip replacement and intramedullary nail groups in terms of length of surgery, which is in contrast to the previous study.^9^ The improvements of fixation devices and progress of surgical skills made the greatest contributions to shortening the surgery time.

In our study, we used the Harris hip score to measure the functional outcomes of the hip joint. The result showed intramedullary nail had comparable functional outcomes with hip replacement. One of the included trials reported the Harris hip score at 6 months, while the remaining reported at 12 months. Park^32^ showed that there was no difference between the intramedullary nail and hip replacement until 12 months, yet scores were significantly better in intramedullary nail group when measured at 24 months after the surgery. In another perspective study, Ozkayin *et al*. reported the difference was statistically significant between the patients treated with hemiarthroplasty and PFN in favor of the former within the first 3 months. However, this difference diminished at the 6th month, and even reversed at the 12th month postoperatively.^33^ Thus, trials with a longer follow‐up to evaluate the final outcomes are needed.

The meta‐analysis showed there was no statistical difference between hip replacement and intramedullary nail in terms of mortality. Tang *et al*. concluded PFNA had a significant superiority over hemiarthroplasty regarding the 1‐year mortality, the 3‐year mortality, and total mortality in a retrospective study.^34^ Patients who had a hip fracture were at risk of cardiovascular, pulmonary, thrombotic, infectious, and neurological complications which could result in death. Therefore, the hip replacement could allow the patients to bear weight much earlier than intramedullary nail, which had a notable effect on prevention of medical complications resulted from long‐term immobilization.

Without doubt, there were several limitations in this meta‐analysis. First, the number of studies included in this study was not sufficient. Second, the quality of the trials was poor and, in some trials, the demographic information and fracture type were unclear, which may introduce bias to the results. Third, the included trials had different timetables of follow‐up, and the functional outcomes were assessed at different time point. Furthermore, the existence of publication bias was common to all meta‐analysis.

### *Conclusions*

Based on the meta‐analysis, we found that hip replacement can shorten immobilization time and reduce implant‐related complications and post‐operative complication rates, but it has greater intraoperative blood loss. The meta‐analysis showed there is no obvious statistical differences in terms of length of surgery, number of units transfused, hospital stay, Harris hip score, and mortality. In the absence of solid evidence, the hip replacement should be taken cautiously in carefully selected elderly adults with unstable intertrochanteric fracture. We suggest hip replacement should be considered as a primary treatment option in elderly adults with comorbidities and several risk factors of mechanical and post‐operative complications.

## Implications for Research

These limited studies provide some evidence that hip replacement can achieve comparable results to those of intramedullary nail for unstable trochanteric fractures. Further larger randomized controlled trials comparing two methods of treatment for unstable intertrochanteric fracture in elderly adults, with longer‐term follow up, are required.
